# Effects of Aquatic Exercise on Individuals with Hypertension: A Systematic Review

**DOI:** 10.3390/healthcare14040513

**Published:** 2026-02-17

**Authors:** Hugo Rodríguez-Otero, Pablo Hernandez-Lucas, Isabel Escobio-Prieto, Eva Lantarón-Caeiro

**Affiliations:** 1 Department of Functional Biology and Health Sciences, Faculty of Physiotherapy, Universidade de Vigo, Campus A Xunqueira, 36005 Pontevedra, Spain; hugorodriguezotero@gmail.com (H.R.-O.); evalantaron@uvigo.es (E.L.-C.); 2 Institute of Biomedicine of Seville (IBIS), Department of Physiotherapy, University of Seville, 41902 Seville, Spain; iescobio@us.es; 3 Research Group CTS-1137, Neurological Physiotherapy Innovative Neurorehabilitation and Neurodevelopment Disorders, NEUROPHYSIUS, 41008 Seville, Spain

**Keywords:** exercise therapy, health, hypertension, physical therapy modalities

## Abstract

**Introduction:** Exercise has proven to be an excellent tool for improving health in individuals with hypertension. A particularly interesting environment for performing exercise is the aquatic medium, whose unique properties have shown effectiveness in reducing blood pressure values. **Objective:** Our objective was to provide an update on the available scientific evidence regarding the effects of aquatic exercise on individuals with hypertension. **Methods:** A systematic search was conducted in the databases PubMed, Web of Science, CINAHL, Sport Discus, Medline, Scopus, and PEDro. Methodological quality was assessed using the PEDro scale, and the risk of bias was evaluated using the RoB 2 tool. Results: Eleven studies were included, with a total of 402 participants, obtaining a mean score of 5.7 on the PEDro scale. Five studies presented a high risk of bias, four showed a low risk, and in two, the risk was unclear. The most used therapeutic exercises in the analyzed interventions were water walking, aquatic mobility and strength exercises, aquatic high-intensity interval training, and aquatic calisthenics. Notably, none of the interventions included swimming. **Conclusions:** Aquatic exercise appears to be associated with reductions in blood pressure in individuals with hypertension; however, these findings should be interpreted cautiously due to the methodological limitations and heterogeneity of the included studies.

## 1. Introduction

According to the World Health Organization, hypertension (HTN) is one of the most prevalent cardiovascular diseases worldwide, affecting a total of 1.28 billion people aged between 30 and 79, and being responsible for over 8.5 million deaths globally [1]. Additionally, having HTN is a risk factor for stroke, myocardial infarction, heart failure and chronic kidney disease at any age, as well as worsening quality of life and increasing healthcare costs [2].

Due to the high prevalence and negative consequences of HTN, healthcare professionals are compelled to seek alternatives to pharmacological treatment [3]. Physical inactivity has significantly contributed to the exponential rise in the number of individuals affected by HTN. To address this issue, evidence shows that increasing physical activity and participating in regular exercise can achieve effects comparable to, or even greater than, pharmacological treatments, without the associated side effects [4,5,6,7].

Recent systematic reviews have reinforced this idea in individuals with HTN, showing reductions in resting blood pressure (BP) and arterial resistance [8,9]. A highly beneficial form of exercise is aquatic exercise, which enables activities to be performed with reduced gravitational impact, provides resistance through water, and allows for high-intensity workouts with lower perceived effort [10]. Additionally, the aquatic environment offers specific benefits for patients with HTN, including suppression of the sympathetic nervous system and a reduction in vascular resistance [11]. From a cardiovascular standpoint, the aquatic environment could be favourable, in addition to the well-known benefits of physical exercise. It is worth mentioning that aquatic exercise has shown positive effects in multiple systematic reviews, such as reducing fatigue in women with cancer [12], improving balance and spasticity in patients who have suffered a stroke [13], improving symptoms in people with depression [14], as well as the lipid profile and BP of individuals with diabetes mellitus [10]. Additionally, a review compiling articles up to 2017 suggests the effectiveness of aquatic exercise in reducing BP [15]. However, the effects and benefits of aquatic exercise are still being investigated today [16]. For these reasons, and because the prevalence of HTN continues to rise [17], the objective of this systematic review is to update and analyze the effects of aquatic exercise on individuals with HTN.

## 2. Materials and Methods

### 2.1. Design

This systematic review followed the Preferred Reporting Items for Systematic Reviews and Meta-Analyses (PRISMA [18]) guidelines and the recommendations for their implementation in exercise, rehabilitation, and sport sciences (PERSiST [19]). It was registered in PROSPERO (registration code: CRD42024549899). The systematic search was conducted during April 2024 across seven databases: PubMed, Web of Science, CINAHL, Sport Discus, Medline, Scopus, and PEDro. The search equation included a combination of MESH terms (aquatic therapy and hypertension) and free terms, with or without truncation (using the asterisk), which provided greater variability: (aquatic aerobics, aquatic exercise, aquatic sport*, aquatic rehabilitation, aquatic activity, aquatic physical therapy, water-based exercise*, water aerobics, water exercise*, water sport*, water rehabilitation, water activity, water therapy, swimming) with Boolean operators AND and OR (Supplementary Material Table S1).

The search strategy, based on the PICOS framework, was as follows: P—population: individuals over 18 years old with HTN; I—intervention: aquatic exercise; C—comparison: another intervention, placebo, or no intervention; O—outcomes: any health variable related to hypertension (HTN) and the cardiorespiratory system, such as effects on blood pressure (BP), maximum oxygen uptake (VO2Max), and endothelial function; S—study design: randomized controlled trials.

### 2.2. Study Selection

First, duplicate articles were removed. The two most experienced authors in cardiac rehabilitation conducted the study selection process to ensure expert evaluation. A third reviewer resolved any discrepancies. The inter-reviewer agreement was assessed using the Kappa index, yielding a value of 0.9, indicating near-perfect agreement. Once this process was completed, each study was analyzed using the following inclusion criteria: (i) individuals over 18 years old, (ii) with HTN, (iii) randomized controlled trials (RCTs), (iv) intervention involving aquatic exercise, (v) publication in English or Spanish. Exclusion criteria were also applied: (i) full text not available, (ii) publication date earlier than 2017. The exclusion criteria were applied using a customized Microsoft Excel table to filter the results. The restriction to studies published from 2017 onwards was applied to provide an update of the most recent evidence, as the earlier literature on aquatic exercise and blood pressure had already been synthesized in a previous systematic review [15].

### 2.3. Data Extraction

The following data were extracted for further analysis: demographic information (title, authors, journal, and year), sample characteristics (age, sex, number of participants, inclusion/exclusion criteria, and supervisor), specific study parameters (duration of intervention, adverse events, and exercise methods), and the outcomes obtained (analyzed variables, instruments used, and follow-up duration). Tables were used to describe both the study characteristics and the extracted data.

### 2.4. Quality Assessment

The PEDro scale [20] was used to assess the methodological quality of the studies, and the Risk of Bias 2.0 tool (RoB 2) [21] was applied to analyze the risk of bias, differentiating between parallel and crossover studies. Two independent reviewers applied the respective scales to assess the validity of the articles. In cases of disagreement, a third reviewer was consulted to make the final decision on the studies included in the analysis.

### 2.5. Data Synthesis

Due to substantial heterogeneity among the included studies in terms of intervention duration, frequency, intensity, protocol design, outcome measures, and the predominance of studies assessing acute responses, a quantitative meta-analysis was not considered appropriate. Therefore, a structured qualitative synthesis was conducted. The findings were organized according to the type of intervention (acute responses vs. structured training programmes) and the cardiovascular outcomes evaluated.

## 3. Results

### 3.1. Study Selection

**Figure 1:**
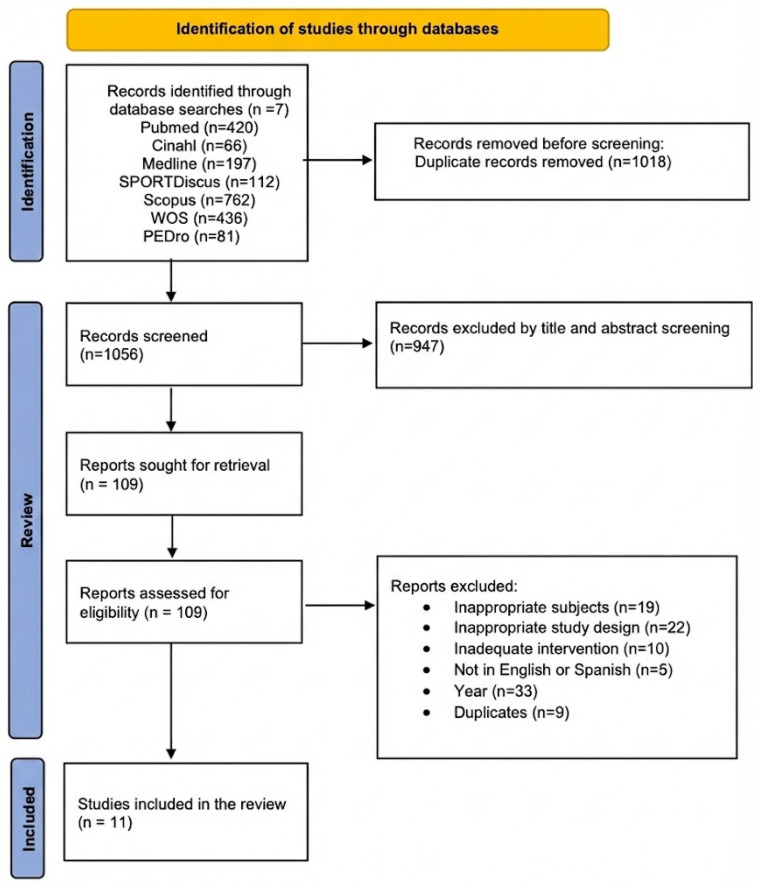
PRISMA flow diagram. The colored side bars are used only for visual grouping of the phases (Identification, Review, Included) and do not encode additional data.

From the 2074 results in the initial search, 1056 articles were identified for analysis after removing duplicates. Through title and abstract screening, 947 articles were excluded for not meeting the inclusion criteria. Subsequently, the remaining 109 articles were subjected to exclusion criteria using a customized Microsoft Excel template. At the full-text stage, the most frequent reasons for exclusion were non-randomized study design, interventions not involving aquatic exercise, populations without hypertension, absence of relevant cardiovascular outcomes, and duplicate reporting of results. Finally, 11 articles were included in this review [22,23,24,25,26,27,28,29,30,31,32]. Figure 1 shows the flow diagram detailing the study selection process.

### 3.2. Methodological Quality and Risk of Bias in the Included Studies

The methodological quality of the studies was 4 points or higher. The highest score was 7 [29,30]. The average score of the articles was 5.7 (Table 1) [33].

**Figure 2:**
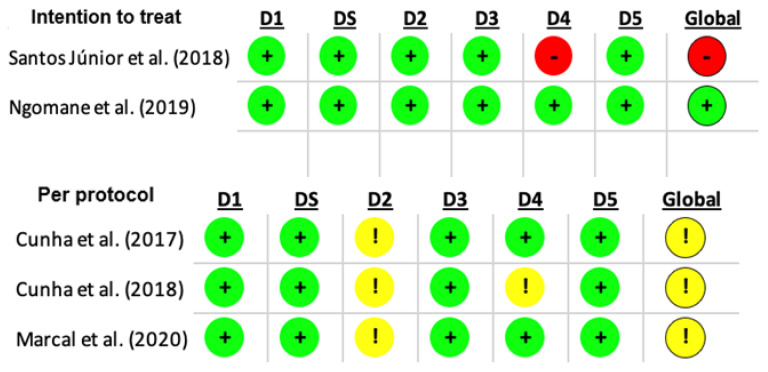
Risk of bias assessment using the RoB 2 tool for crossover studies. Domains: D1, randomization process; D2, deviations from intended interventions; D3, missing outcome data; D4, outcome measurement; D5, selection of the reported results. Color coding: red = high risk of bias; yellow = some concerns; green = low risk of bias [24,25,27,29,30].

**Figure 3:**
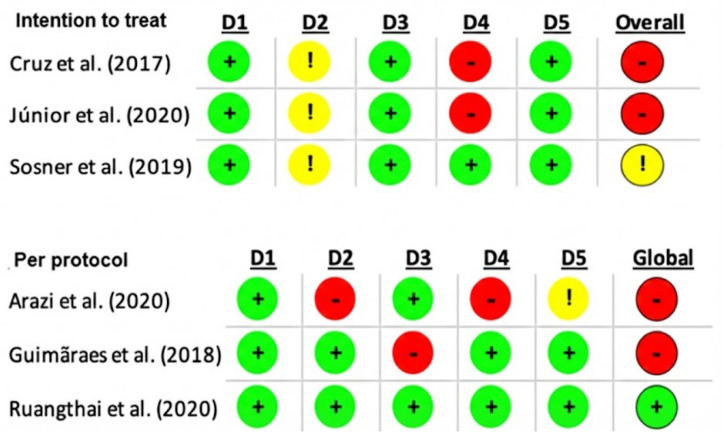
Risk of bias assessment using the RoB 2 tool for parallel studies. Domains: D1, randomization process; D2, deviations from intended interventions; D3, missing outcome data; D4, outcome measurement; D5, selection of the reported result. Color coding: red = high risk of bias; yellow = some concerns; green = low risk of bias [22,23,26,28,31,32].

In all studies, there was no selection bias. Regarding the parallel RCTs [22,23,26,28,31,32], the most common bias was the outcome measurement bias [22,23,28] (Figure 2 and Figure 3).

### 3.3. Sample Characteristics

The age of participants in most studies was over 60 years [24,25,27,28,29,30,31]. Only three studies included individuals under 55 years of age [23,26,32]. Two studies included only women [22,28] (Table 2).

The total sample analyzed across the 11 included studies comprised 402 participants, of whom 185 engaged in aquatic exercise, 55 participated in dry-land training, and 162 were allocated to the control group. In terms of BP levels, the minimum inclusion criterion in all studies was ≥130/85 mmHg. However, in 63.6% of the studies, participants were required to have BP levels of ≥140/90 mmHg to be included in the study [23,24,25,26,27,29,30] (Supplementary Material Table S2). No adverse effects were reported in any of the studies included in the systematic review [22,23,24,25,26,27,28,29,30,31,32].

### 3.4. Characteristics of the Intervention

The interventions analyzed showed considerable variability in design, ranging from single-session protocols assessing acute responses to multi-week programmes aimed at inducing training adaptations. In 54.5% of the studies, a single aquatic exercise session was conducted, and the hypotensive response and BP behavior were assessed over the 24 h post-session [24,25,27,28,29,30]. Additionally, five studies conducted an intervention consisting of two or three weekly sessions over 2, 8, or even 12 weeks [22,23,26,31,32]. Session duration generally ranged from 40 to 60 min [22,23,24,25,26,27,28,31], except for three studies where it was 30 min [29,30,32] (Table 2).

The aquatic exercise sessions were divided into a 5–10 min warm-up, a main part lasting between 30 and 40 min, and a 5–10 min cool-down in all studies [22,23,24,25,26,27,28,29,30,31], except for one study where the main part lasted for 20 min [32]. Most sessions consisted of aerobic exercises (walking or running) combined with strength-resistance exercises against the water (moving different body segments, weight transfers, and/or functional movements) [22,23,24,25,27,28,31]. Notably, none of the interventions included swimming.

To calculate exercise intensity, most studies used maximum heart rate [22,24,25,27], the Rate of Perceived Exertion scale [29,30] or the Borg scale [23,26,31]. In two other studies, Peak Power Output [32] and heart rate reserve [28] were used.

In all the studies analyzed, the water temperature was around 30 °C [23,24,25,26,27,28,29,30,31,32], except for the study by Arazi et al. [22], where the temperature ranged from 34 to 36 °C.

Regarding the control groups, in three studies [22,23,26], participants were instructed to maintain their daily life activities. In two studies, water immersion was performed [27,30]. In four studies, participants remained seated or standing in conditions similar to the intervention group [24,25,29]. In two studies, they performed exercise. And in one study, the control condition was not specified [31] (Supplementary Material Table S3).

### 3.5. Results of the Studies Provided in Relation to BP, VO2max, and Endothelial Function

Studies assessing acute post-exercise responses reported BP changes within 24 h following aquatic exercise sessions. In contrast, studies evaluating multi-week training programmes reported reductions in resting BP measured at post-intervention time points. Most included studies reported reductions in BP following aquatic exercise, although the timing and magnitude varied across protocols and assessment windows [22,23,24,25,26,27,28,29,30,31,32]. Only a limited number of training-based studies assessed cardiorespiratory fitness; among those, improvements in VO_2_max were reported in some trials, while others did not observe significant changes. Specifically, Arazi et al. [22] reported a significant increase in the aquatic exercise group compared with baseline values. However, in the studies by Júnior et al. [27] and Ruangthai et al. [31], no significant improvements in this variable were observed in any of the groups. Endothelial function and oxidative stress markers were assessed in fewer studies, with some studies reporting significant changes and others reporting no significant differences. As for endothelial function, the studies by Ngomane et al. [29] and Marcal et al. [30] did not report significant changes in endothelial reactivity in the groups that performed aquatic exercise compared to other groups. Nonetheless, Ruangthai et al. [31] found significant improvements in oxidative stress markers and endothelial function in the aquatic exercise group compared to the control and land-based exercise groups (Table 2).

## 4. Discussion

The purpose of this systematic review was to evaluate the effects of aquatic exercise on individuals with HTN. The main findings indicate that aquatic exercise may contribute to reductions in BP levels [22,23,24,25,26,27,28,29,30,31,32], and may also be associated with improvements in cardiorespiratory fitness, such as VO_2_max [22,31] and cardio metabolic parameters [31]. It is important to distinguish between statistical significance and clinical relevance. While several studies demonstrated significant reductions in blood pressure, the clinical impact of these changes may differ depending on their magnitude and whether they represent acute responses or sustained adaptations. Even modest reductions in blood pressure have been associated with reduced cardiovascular risk at the population level; however, the heterogeneity of protocols and predominance of acute studies in this review limit direct clinical extrapolation. More consistent reductions in blood pressure were observed in studies involving structured multi-week programmes that combined aerobic and resistance exercise in thermoneutral water, particularly among participants with moderate hypertension. In contrast, single-session interventions produced more variable responses, reflecting the transient nature of acute post-exercise hypotension. Differences in baseline BP levels may also have influenced the magnitude of change, with participants presenting higher initial BP values showing greater absolute reductions.

Regarding structured exercise, the results of this review are consistent with the effects of exercise on BP found in other reviews, showing a reduction in BP [9,34]. Performing exercise, regardless of its intensity, has been shown to reduce the risk of hypertensive incidents and improve endothelial pathophysiology in individuals with HTN [34]. In HTN, the endothelium can experience dysfunction, leading to several adverse changes: decreased nitric oxide production, increased production of reactive oxygen species, inflammation, and procoagulation [35]. Multiple reviews have demonstrated positive effects in individuals with HTN, regardless of the type of exercise (aerobic, resistance, or isometric), age, sex, or ethnicity [36,37,38].

Most reviews have evaluated the effects of exercise on hypertension primarily on land [36,37,38]. However, the aquatic environment offers multiple advantages over land-based exercise, allowing individuals with comorbidities to engage in physical activity [39]. Additionally, energy expenditure in water is similar to that of land-based exercise [40]. In recent years, aquatic exercise has gained popularity due to its unique benefits, such as the natural resistance of water, which enables low-impact yet effective training for muscle strengthening and the management of chronic conditions [10]. Furthermore, it reduces body weight and joint stress, making it accessible to most individuals [41,42]. Additionally, Zhou et al. [16] highlight that, due to these benefits, research on aquatic exercise and its impact on cardiovascular health has increased. Angraini et al. [43], in a broader review on hydrotherapy, suggest that aquatic exercise could significantly reduce blood pressure in patients with hypertension. Similarly, a 2018 review, which included both healthy adults and individuals with hypertension, reported comparable benefits [37].

The effectiveness of exercise in lowering BP may be greater in aquatic environments compared to land-based exercise, likely due to the physiological changes induced by partial body immersion, such as reduced vascular tone and peripheral vascular resistance [11]. These effects may enhance the body’s response to exercise in water compared to land-based exercise [11]. Water immersion affects the cardiovascular system by redistributing blood flow to the heart due to hydrostatic pressure gradients [44,45]. This may increase cardiac output and contribute to a hypotensive response, which can also be influenced by water temperature [44,45]. However, it should be noted that most of these mechanistic explanations are based on established physiological principles and the previous literature, as only a limited number of included studies directly assessed endothelial or oxidative stress markers.

Water immersion alone could trigger a hypotensive response [46]. This may partly explain the variability observed among the included studies. For example, Marçal et al. [30] did not observe significant BP changes compared to the control condition despite 30 min of water immersion, whereas Santos Júnior et al. [27] reported significant hypotensive responses under similar immersion durations [27].

When performing exercise in water, it is important to consider water temperature, as studies have shown that the water’s temperature can produce different physiological effects on the body [45,47,48]. For example, water slightly cooler than body temperature reduces overheating, warm water may relieve joint pain, and cold water lowers heart rate and/or cardiac output [45,47,48]. Additionally, temperatures around 30 °C influence metabolism due to increased sympathetic system activation [49]. Generally, recommendations for moderate-to-vigorous intensity aquatic exercise in elite athletes suggest maintaining water temperatures between 25 °C and 28 °C [50]. However, other authors recommend that the water temperature be neutral, between 33.5 °C and 35.5 °C [44]. This allows for sufficiently prolonged immersion periods for exercise in various pathologies [44]. In the studies analyzed, the water temperature in all but one study was around 30 °C, except for the study by Arazi et al. [22], where the temperature ranged from 34 to 36 °C, as the study was conducted in volcanic waters. Higher than recommended temperatures have been suggested to potentially influence the hypotensive response [51].

When it comes to the type of exercise used, the included studies utilized aerobic and/or resistance training against water [22,23,24,25,27,28,31] at a moderate intensity. However, a meta-analytic review suggests that the most effective type of exercise for reducing systolic and diastolic BP is isometric exercise [9]. This may be due to its acute activation of the metaboreflex, which reduces oxidative stress in tissues, improves endothelial function, and promotes changes in baroreflex sensitivity, as well as long-term balance in the autonomic nervous system [52]. Some of these effects might also be achieved through prolonged aquatic exercise [53]. Nonetheless, it would be interesting to evaluate BP responses to an aquatic exercise program incorporating isometric exercise.

The intensity used in most studies was moderate [22,23,25,26,28,29,30,31], except for the study by Santos Júnior et al. [27], where they reached up to 89% of maximum heart rate, and Sosner et al. [32], where they achieved 100% of peak oxygen power. In the latter, a greater reduction in BP was observed within 24 h post-exercise compared to the moderate exercise group [32]. However, it is important to note that, in the review conducted by Caldas Costa et al. [54], both the moderate-intensity exercise group and the high-intensity group achieved similar reductions in BP. These findings were supported by a later review by Leal et al. [55].

Regarding the duration of the interventions, only five studies [22,23,26,31,32] included multiple sessions to promote chronic adaptations rather than acute hypotensive responses. Long-term BP reduction may result from improved baroreflex sensitivity, achievable after ~8 weeks of training [53,56]. However, these mechanistic interpretations are based on physiological rationale and the previous literature, as most included studies did not directly measure these pathways.

Analyzing the studies that aimed to assess changes in VO2max [22,23,28], significant improvements were only observed in the study by Arazi et al. [22]. This may be related to the fact that detectable improvements in VO_2_max generally require interventions lasting at least 4–8 weeks [57]. Among the studies analyzed, only two reached the minimum duration of 4 weeks [22,23].

Several studies [22,23,24,25,32] have reported 100% adherence to the exercise program [22,23,24,25,32]. This may be due to the fact that aquatic exercise is enjoyable, provides a change in environment, and results in a lower perceived effort compared to land-based exercise [10,58].

This review presents several methodological limitations that should be considered when interpreting the findings. The included studies showed marked variability in intervention characteristics, such as duration (single-session vs. multi-week programmes), frequency, intensity, water temperature, and outcome measures, which limits direct comparisons and reduces the generalizability of the results. Furthermore, the predominance of studies assessing acute post-exercise responses, compared with fewer trials evaluating structured training interventions, complicates interpretation of sustained effects. The absence of long-term follow-up data and the lack of meta-analytic synthesis further constrain the strength of the conclusions.

In addition, the overall methodological quality of the studies was moderate, with several trials presenting a high risk of bias, particularly in outcome measurement and blinding procedures, which are common challenges in exercise research. The relatively small number of eligible studies, together with the limited number of randomized controlled trials, reduces the overall certainty of the evidence. The restriction to publications from 2017 onwards may have excluded relevant earlier trials and introduced temporal bias. Similarly, restricting inclusion to studies published in English or Spanish may have excluded relevant evidence in other languages and represents a potential source of language bias. The possibility of publication bias cannot be ruled out, as studies reporting positive findings are more likely to be published. These methodological issues restrict the strength of the conclusions and limit the generalizability of the findings to broader hypertensive populations. Future research should prioritize well-designed longitudinal trials to better clarify long-term adaptations and underlying physiological mechanisms.

## 5. Conclusions

Aquatic exercise appears to be a potentially effective and safe alternative for reducing blood pressure in individuals with hypertension, although these findings should be interpreted with caution given the moderate methodological quality and risk of bias identified in several included studies. This systematic review analyzed eleven studies with 402 participants, where the interventions included water walking, mobility exercises, strength training, and high-intensity interval training. The results show a significant reduction in blood pressure compared to control groups, along with improvements in VO_2_max and oxidative stress markers. These effects may be partly explained by the reduction in peripheral vascular resistance induced by immersion, along with lower perceived effort and a change in environment, which enhance exercise adherence.

Most studies included patients with moderate to high hypertension, although some considered mild cases, suggesting benefits across different disease severities. However, it is important to note that most of the available evidence relates to acute post-exercise responses, while only a smaller number of studies investigated structured programmes lasting several weeks. Therefore, evidence supporting chronic cardiovascular adaptations remains preliminary. Due to variability in protocols and lack of follow-up, more rigorous and prolonged trials are needed to consolidate the evidence and define an optimal aquatic exercise program.

## Figures and Tables

**Table 1:** Methodological quality according to the PEDro scale.

Author	1 *	2	3	4	5	6	7	8	9	10	11	Score
Arazi et al. [22]	1	1	0	1	0	0	0	1	0	1	1	5/10
Cruz et al. [23]	1	1	1	1	0	0	0	1	1	1	1	7/10
Cunha et al. [24]	1	1	0	1	0	0	0	0	1	1	1	5/10
Cunha et al. [25]	1	1	1	1	0	0	0	0	1	1	1	6/10
Guimãraes et al. [26]	0	1	1	1	0	0	1	0	0	1	1	6/10
Santos Júnior et al. [27]	1	1	1	1	0	0	0	0	0	1	1	5/10
Júnior et al. [28]	1	1	0	1	0	0	0	0	1	1	1	5/10
Marcal et al. [29]	1	1	1	1	0	0	1	0	1	1	1	7/10
Ngomane et al. [30]	1	1	1	1	0	0	1	1	0	1	1	7/10
Ruangthai et al. [31]	1	1	0	1	0	0	0	0	0	1	1	4/10
Sosner et al. [32]	1	1	0	1	0	0	0	1	1	1	1	6/10

It shows a 1 if the item is present and a 0 if not. Items: (1) specified selection criteria; (2) random allocation; (3) concealed allocation; (4) baseline comparability; (5) blinding of subjects; (6) blinding of therapists; (7) blinding of assessors; (8) outcomes greater than 85%; (9) intention-to-treat analysis; (10) group comparisons; (11) measures of data and variability. * This item relates to external validity and is therefore not considered in the final score.

**Table 2:** Characteristics of the intervention in the analyzed studies.

Author	Sample (Women)	Intervention	Duration	Frequency of Intervention	Results
Arazi et al. [22]	58.55 (100%)	G1: AE (n = 10) G2: CG (n = 10)	8 weeks	2 sessions per week, 50–60 min per session	G1 had significant improvements in systolic BP, body mass percentage, and VO2max compared to pre-test. The systolic BP decreased from 134.12 ± 32.89 mmHg to 116.97 ± 19.50 mmHg (−17.15 mmHg), whereas in G2, it remained unchanged (+1.66 mmHg, NS). No significant changes in diastolic BP were found.
Cruz et al. [23]	53.3 (47.7%)	G1: AE (n = 28) G2: CG (n = 16)	12 weeks	3 sessions per week, 60 min per session	G1 showed a significant decrease in systolic BP from 162.2 ± 23.2 mmHg to 135.5 ± 11 mmHg, while G2 remained unchanged (157.6 ± 17.6 mmHg to 157.8 ± 16.6 mmHg).
Cunha et al. [24]	67.8 (100%)	G1: AE (n = 25) G2: CG (n = 25)	3 days	1 sessions per week, 45 min per session	G1 had a transient increase in systolic BP of 17.4 mHg (14.3%) immediately post-exercise, followed by a greater BP decline at 10 min (SBP: −7.5 mmHg; DBP: −3.8 mmHg). BP stabilized at 20 min post-exercise. G1 and G2 did not achieve major responses within the rest of the parameters.
Cunha et al. [25]	70 (100%)	G1: AE (n = 27) G2: CG (n = 27)	1 week	1 sessions per week, 45 min per session	G1 showed a decrease in systolic BP of 5.1 ± 1.0 mmHg over 21 h, with reductions of 5.7 ± 1.1 mmHg during waking hours and 4.5 ± 0.4 mmHg during sleep. Diastolic BP decreased slightly (1.2 ± 0.3 mmHg over 21 h). G2 did not achieve major responses in any parameter.
Guimãraes et al. [26]	53 (53.13)	G1: AE (n = 16) G2: CG (n = 16)	12 weeks	3 sessions per week, 60 min per session	G1 had significant reductions in 24 h systolic BP (−19.5 ± 4.6 mmHg) and diastolic BP (−11.1 ± 2.4 mmHg) at week 12, which persisted at week 24 but attenuated (SBP: −9.6 ± 3.8 mmHg; DBP: −7.5 ± 2.2 mmHg). G2 did not achieve major responses in any parameter.
Santos Júnior et al. [27]	67.3 (100%)	G1: AE (n = 12) G2: CG (n = 12)	1 week	1 sessions per week, 45 min per session	G1 had a significant post-exercise reduction in systolic BP at all time points, from 130.1 ± 13.3 mmHg at rest to 115.1 ± 11.8 mmHg at 15 min, 114.9 ± 11.8 mmHg at 30 min, 112.0 ± 13.1 mmHg at 45 min, and 115.7 ± 14.7 mmHg at 60 min. No significant changes in diastolic BP were found.
Júnior et al. [28]	66.5 (100%)	G1: AE (n = 10) G2: AE (n = 10) G3: LE (n = 10) G4: LE-PEH (n = 10)	3 days	1 sessions per week, 50 min per session	G1 had significantly lower systolic BP (160 mmHg [150.9–191.7]) versus LE (162.5 mmHg [151.3–177.9]), and a significantly lower diastolic BP in WAT (80.0 mmHg [72.4–89.6]) vs. LE (90.0 mmHg [84.1–90.5]).
Marcal et al. [29]	67 (100%)	G1: AE (n = 11) G2; AE (n = 11) G3: CG (n = 11)	24 h	1 sessions per week, 30 min per session	The HIIE group showed a minimal reduction in systolic BP of 3–5 mmHg and diastolic BP of 1–3 mmHg, while the moderate-intensity aerobic exercise (MICE) group showed no relevant changes.
Ngomane et al. [30]	66.4 (60%)	G1: AE (n = 15) G2: LE (n = 15) G3: CG (n = 15)	1 week	1 sessions per week, 30 min per session	G1 had a significant systolic BP reduction (−9.9 ± 3.1 mmHg) at 45 min post-exercise and improved 24 h BP (systolic −9.5 ± 3.0 mmHg, diastolic −4.5 ± 1.3 mmHg). There were no significant changes in arterial resistance or in endothelial reactivity.
Ruangthai et al. [31]	69.2 (78.46)	G1: AE (n = 17) G2: LE (n = 16) G3: CG (n = 20)	12 weeks	3 sessions per week, 60 min per session	G1 and G2 significantly improved systolic BP (−11.6 mmHg [8.2%] in LET, −6.5 mmHg [4.6%] in WET). G1 and G2 significantly improved, rate-pressure product, GPx, NOx-, MDA, hs-CR concentrations, physical and psychological domains and quality of life. G2 improved the combined index of LDL-C and lipoproteins. G2 improved the Sit to Stand Test and the 2 min Step Test. No significant changes were observed within the rest of the parameters.
Sosner et al. [32]	65 (48%)	G1: AE (n = 14) G2: AE (n = 14) G3: LE (n = 14)	2 weeks	3 sessions per week, 20–30 min per session	G1 significantly reduced to 24 h SBP (−5.1 mmHg) and DBP (−2.9 mmHg), with similar reductions in daytime BP (SBP: −6.2 mmHg; DBP: −3.4 mmHg). G2 and G3 did not improve arterial resistance significantly.

AE: aquatic exercise; CG: control group; BP: blood pressure; VO2max: maximum oxygen volume; SBP: systolic blood pressure; DBP: diastolic blood pressure; ABPM: ambulatory blood pressure monitoring; PEH: post-exercise hypotensive response; LE: land-based exercise; HIIE: high-intensity interval exercise; MICE: moderate-intensity continuous exercise; HRV: heart rate variability; GPx: glutathione peroxidase; NOx-: total nitrites/nitrates; MDA: malondialdehyde; hs-CRP: high-sensitivity C-reactive protein.

## Data Availability

No new data were created or analyzed in this study. Data sharing is not applicable to this article.

## Supplementary Materials

The following supporting information can be downloaded at https://www.mdpi.com/article/10.3390/healthcare14040513/s1: Table S1. Search equations according to the PICOS question; Table S2. Age range, inclusion and exclusion criteria; Table S3. Summary of exercise.

## Abbreviations

The following abbreviations are used in this manuscript:

BP	Blood Pressure
CINAHL	Cumulative Index to Nursing and Allied Health Literature
EAPC	European Association of Preventive Cardiology
ESC	European Society of Cardiology
HIIT	High-Intensity Interval Training
HTN	Hypertension
MeSH	Medical Subject Headings
MET	Metabolic Equivalent of Task
PEDro	Physiotherapy Evidence Database
PERSiST	PRISMA in Exercise, Rehabilitation, and Sport Sciences
PICOS	Population, Intervention, Comparison, Outcomes, Study design
PRISMA	Preferred Reporting Items for Systematic Reviews and Meta-Analyses
PROSPERO	International Prospective Register of Systematic Reviews
RCT	Randomized Controlled Trial
RoB 2	Risk of Bias 2.0
VO_2_max	Maximum Oxygen Uptake
