# Supplementary material for: Effects of Aquatic Exercise on Individuals with Hypertension: A Systematic Review
Source: Healthcare (Basel). 2026 Feb 17;14(4):513. doi: 10.3390/healthcare14040513 (PMC12941251; doi:10.3390/healthcare14040513)
Supplement: Supplementary file 1 [file healthcare-14-00513-s001.zip › Suplementary1.pdf]

Supplementary Material Table 1. Search equations according to the PICOS question.

Database	**Search equations**
Pubmed	("Aquatic Therapy" [Mesh] OR "aquatic aerobics" or "aquatic exercise*" or "aquatic sport*" or "aquatic rehabilitation" or "aquatic activity" or "aquatic physical therapy" or "water-based exercise*" or "water aerobics" or "water exercise*" or "water sport*" or "water rehabilitation" or "water activity" or "water therapy" or "swimming") AND ("Hypertension" [Mesh] OR "hypertension" OR "high blood pressure" OR "hypertensive")
WOS	TS= (("Aquatic Therapy" OR "aquatic aerobics" or "aquatic exercise*" or "aquatic sport*" or "aquatic rehabilitation" or "aquatic activity" or "aquatic physical therapy" or "water-based exercise*" or "water aerobics" or "water exercise*" or "water sport*" or "water rehabilitation" or "water activity" or "water therapy" or "swimming") AND ("hypertension" or "hypertensive" or "high blood pressure"))
Scopus	TITLE-ABS-KEY (("Aquatic Therapy" OR "aquatic aerobics" OR "aquatic exercise*" OR "aquatic sport*" OR "aquatic rehabilitation" OR "aquatic activity" OR "aquatic physical therapy" OR "water-based exercise*" OR "water aerobics" OR "water exercise*" OR "water sport*" OR "water rehabilitation" OR "water activity" OR "water therapy" OR "swimming")) AND TITLE-ABS-KEY ("hypertension" OR "hypertensive" OR "high blood pressure")
Medline	MH (("Aquatic Therapy" OR "aquatic aerobics" or "aquatic exercise*" or "aquatic sport*" or "aquatic rehabilitation" or "aquatic activity" or "aquatic physical therapy" or "water-based exercise*" or "water aerobics" or "water exercise*" or "water sport*" or "water rehabilitation" or "water activity" or "water therapy" or "swimming")) AND ("hypertension" OR "hypertensive" or "high blood pressure")
CINAHL	MH (("Aquatic Therapy" OR "aquatic aerobics" or "aquatic exercise*" or "aquatic sport*" or "aquatic rehabilitation" or "aquatic activity" or "aquatic physical therapy" or "water-based exercise*" or "water aerobics" or "water exercise*" or "water sport*" or "water rehabilitation" or "water activity" or "water therapy" or "swimming")) AND ("hypertension" OR "hypertensive" or "high blood pressure")
Sport Discus	(DE "WATER aerobics" OR DE "WATER bikes" OR DE "SWIMMING" OR DE "AQUATIC exercise therapy" OR DE "AQUATIC exercises" OR "Aquatic Therapy" OR "aquatic aerobics" or "aquatic exercise*" or "aquatic sport*" or "aquatic rehabilitation" or "aquatic activity" or "aquatic physical therapy" or "water-based exercise*" or "water aerobics" or "water exercise*" or "water sport*" or "water rehabilitation" or "water activity" or "water therapy" or "swimming") AND (DE "HYPERTENSION" OR "hypertension" OR "hypertensive" or "high blood pressure")
PEDro	"hydrotherapy, balneotherapy" (therapy) and "cardiothoracics" (subdiscipline)<br>"Aquatic" and "hypertension"<br>"Aquatic exercise and hypertension"<br>"Water" and "hypertension"

"Water exercise" and "hypertension"
"Swimming" and hypertension"
