# Supplementary material for: Effects of Aquatic Exercise on Individuals with Hypertension: A Systematic Review
Source: Healthcare (Basel). 2026 Feb 17;14(4):513. doi: 10.3390/healthcare14040513 (PMC12941251; doi:10.3390/healthcare14040513)
Supplement: Supplementary file 1 [file healthcare-14-00513-s001.zip › Suplementary2.pdf]

Supplementary Material Table 2. Age range, inclusion and exclusion criteria

Author	Age range of participants	Inclusion criteria	Exclusion criteria
Arazi et al. (2018)	55-65	Postmenopausal women under cardiologist supervision with a systolic blood pressure of at least ≥ 130 mmHg or diastolic ≥ 90 mmHg.	Chronic diseases (musculoskeletal or renal) limiting protocol completion, regular physical activity in the 6 months prior to the program, or changes in medication dosage during the program.
Cruz et al. (2017)	40-65	Participants diagnosed with hypertension for more than 5 years who had not changed their medication in the previous 6 months and were using at least three types of medication. They had a systolic blood pressure of at least ≥ 140 mmHg or diastolic ≥ 90 mmHg.	History of secondary hypertension, smokers, diabetes, coronary artery disease, infarction, or any chronic disease limiting exercise capacity. Also excluded were individuals who regularly engaged in physical activity, were unable to complete the cardiopulmonary test, had low adherence to medication, or severe obesity.
Cunha et al. (2017)	65-80	Participants diagnosed with systemic hypertension with a systolic blood pressure of at least ≥ 160 mmHg or diastolic ≥ 100 mmHg, had been practicing aquatic aerobic exercise for at least 6 months, and met the age requirement.	Hyperreactive individuals (systolic BP >220 mmHg and/or diastolic >150 mmHg), fever or infections, insulin use, type 2 or higher obesity, heart failure, recent cardiovascular event, chronic kidney failure, active smoking, orthopedic problems, or physical/psychological limitations preventing exercise.
Cunha et al. (2018)	65-80	Participants diagnosed with hypertension with a systolic blood pressure of at least ≥ 160 mmHg or diastolic ≥ 100 mmHg, physically active, and met the age requirement.	Fever or infections, insulin use, type 2 or higher obesity, heart failure, recent cardiovascular event, chronic kidney failure, active smoking, orthopedic problems, or physical/psychological limitations preventing exercise.
Guimarães et al. (2018)	40-65	Participants diagnosed with hypertension for more than 5 years who had not changed their medication in the previous 6	History of secondary hypertension, smokers, diabetes, coronary artery disease, infarction, or any chronic disease limiting exercise capacity.

		months and were using at least three types of medication. They had a systolic blood pressure of at least ≥ 140 mmHg or diastolic ≥ 90 mmHg.	Also excluded were individuals who regularly engaged in physical activity, had low adherence to medication, or severe obesity.
Santos Júnior et al. (2018)	>60	Diagnosed with hypertension with a systolic blood pressure of at least ≥ 140 mmHg or diastolic ≥ 90 mmHg.	BMI ≥ 30 kg/m² and individuals with diabetes mellitus.
Júnior et al. (2020)	>60	Hypertensive women under medication with an exercise frequency of at least twice per week for at least 6 months before the intervention, with 20 subjects in water and 20 on land.	Cardiorespiratory symptoms, cardiac disorders, metabolic syndromes, renal or hepatic diseases, cognitive impairments, or any contraindications for exercise.
Marcal et al. (2022)	≥60	Individuals diagnosed with hypertension at least 6 months prior without medication changes in a 3-month period, with blood pressure below 140/90 mmHg, and had not participated in structured physical activity or were classified as physically inactive according to the International Physical Activity Questionnaire.	Uncontrolled cardiovascular, respiratory, or metabolic diseases, musculoskeletal disorders, tobacco or alcohol abuse, and physical or mental disabilities.
Ngomane et al. (2018)	>60	Hypertensive individuals under medication for at least 6 months, BP > 150/90 mmHg, without changes in medication in the last 2 months, sedentary lifestyle according to the International Physical Activity Questionnaire, and had not engaged in regular physical activity in the last 3 months.	Uncontrolled cardiovascular, respiratory, or metabolic diseases, musculoskeletal disorders, tobacco or alcohol abuse, physical or mental disabilities, and individuals whose medication was modified during the study.
Ruangthai et al. (2020)	>60	Individuals with hypertension (≥ 130 mmHg systolic or diastolic ≥ 80 mmHg).	Unstable/uncontrolled cardiorespiratory disease, musculoskeletal limitations preventing exercise, cancer or low life expectancy, psychiatric disorders, cognitive diseases or dementia, and impaired vision.

Sosner et al. (2019)	43-80	Individuals over 18 years old with BP ≥ 130/85 mmHg.	Any contraindication for high-intensity exercise, any cardiovascular event in the last 12 months, chronic atrial fibrillation, night shift work, or pregnancy.

[2] Abbreviations: BP: Blood pressure; BMI: Body mass index; mmHg: Millimeters of mercury.
