# Supplementary material for: Effects of Aquatic Exercise on Individuals with Hypertension: A Systematic Review
Source: Healthcare (Basel). 2026 Feb 17;14(4):513. doi: 10.3390/healthcare14040513 (PMC12941251; doi:10.3390/healthcare14040513)
Supplement: Supplementary file 1 [file healthcare-14-00513-s001.zip › Suplementary3.pdf]

Supplementary Material Table 3. Summary of exercise.

Authors	Aquatic Exercise Group	Dry-land Training Group	Control Group
Arazi et al. (2018)	Sessions in a volcanic water pool at 34-36°C. 10 minutes of aquatic walking and stretching, 30-40 minutes of main part (walking, leg movements, weight transfers), and 10 minutes cool-down. Intensity: 60%-75% HRmax.	None.	Maintenance of daily life activities.
Cruz et al. (2017)	In a 32°C pool, 5-minute warm-up, 30-40 minutes of calisthenics and walking (11-13 Borg scale), 5-minute cool-down.	None.	Maintenance of daily life activities.
Cunha et al. (2018)	In a pool, 5-minute dynamic warm-up (55%-60% HRmax), 35 minutes of mobility exercises (70%-75% HRmax), and 5-minute cool-down. Exercises included flexion, extension, adduction, and abduction of upper and lower limbs.	None.	Seated or standing without immersion in similar conditions.
Cunha et al. (2018)	5-minute dynamic warm-up (55%-60% HRmax), 35 minutes of exercises at 70%-75% HRmax, and 5-minute cool-down. 18 continuous exercises, each lasting 2 min 30 sec.	None.	Seated or standing without immersion.
Guimarães et al. (2018)	12-week training, 3 weekly sessions of 60 minutes in a 30-32°C pool. Warm-up, calisthenics, walking (11-13 Borg), and cool-down.	None.	Maintenance of daily life activities.
Santos Júnior et al. (2018)	In a 29°C pool, 10-minute warm-up, 30 minutes of aerobic exercises (60%-89% HRmax) such as jumping jacks, horizontal	None.	Standing in water up to the xiphoid process for 45 minutes without movement.

	adduction/abduction, high knees, and front crawl, 5-minute cool-down.		
Júnior et al. (2020)	5-minute warm-up, 20 minutes of aerobic exercise at 75% HR reserve, 20 minutes of strength exercise, 5-minute stretching.	Same protocol as the aquatic group.	Performed the same training but rested 48 hours before blood pressure measurement.
Marcal et al. (2022)	Moderate-intensity group (WATM): 4-minute warm-up (walking at 9 RPE), followed by 26 minutes of walking (11-13 RPE). High-intensity group (WATH): 4-minute warm-up, 21 minutes of high-intensity exercise (1 minute jogging/running at 15-17 RPE, 2 minutes active recovery at 11-9 RPE).	None.	Seated for 30 minutes in a submerged chair.
Ngomane et al. (2018)	In a 29-32°C pool, 5-minute warm-up, 30-minute walking in water (11-13 RPE), and 5-minute cool-down.	Same protocol but performed on land.	Seated in silence for 40 minutes.
Ruangthai et al. (2020)	In a 26-29°C pool, 10-minute mobility warm-up, 20 minutes of aerobic exercise (walking in water, 50-60% HRmax, increased to 60-70% at 6 weeks), 20 minutes of resistance exercise against water (4-5/10 Borg, increased to 6-7/10 at 6 weeks), 10-minute cool-down with stretching.	Same protocol but with resistance training on land (2 sets of 12 reps at 50-70% 1RM, increased to 60-80% in 6 weeks).	Not specified.
Sosner et al. (2019)	Cycle ergometer training in a 30°C pool. 5-minute warm-up at 50% PPO, 2 sets of 10 minutes high-intensity training (15 sec work at 100% PPO, 15 sec passive recovery), 4-minute	Same high intensity cycling protocol but performed on land.	5-minute warm-up at 50 watts, 24 minutes at 50% PPO, and 5-minute cool-down.

rest between sets, 5-minute cool-down.

[3] Abbreviations: CG: Control Group; G1: Group 1; G2: Group 2; G3: Group 3; G4: Group 4; LE: Dry-land Exercise; HR: Heart Rate; HRmax: Maximum Heart Rate; HRres: Heart Rate Reserve; PPO: Peak Power Output; 1RM: One Repetition Maximum; RPE: Rating of Perceived Exertion (6-20 scale); WAT: Aquatic Training Group; WATM: Moderate-Intensity Aquatic Training Group; WATH: High-Intensity Aquatic Training Group.
